# Association of Dietary Animal and Plant Protein Composition with All-Cause Mortality: 24-Year Population-Based Cohort Study

**DOI:** 10.3390/nu18122035

**Published:** 2026-06-22

**Authors:** Federica Prinelli, Antonio Giampiero Russo

**Affiliations:** 1 Institute of Biomedical Technologies, National Research Council, Via Fratelli Cervi 93, 20054 Segrate, MI, Italy; 2 Epidemiology Unit, Agency for Health Protection of the Metropolitan City of Milan, Via Conca del Naviglio, 45, 20123 Milano, MI, Italy; agrusso@ats-milano.it

**Keywords:** dietary proteins, all-cause mortality, compositional data analysis, population-based cohort study

## Abstract

**Background**: This study examined the associations of dietary animal (AP) and plant protein (PP) with all-cause mortality in an Italian population and assessed the potential effect modification by sex, smoking status, and overweight/obesity (defined as BMI ≥ 25 kg/m^2^). **Methods**: This longitudinal population-based study included 1350 adults (50.2% females), aged 40–74 years, recruited between 1991 and 1995, who were followed for all-cause mortality through the regional mortality registry until 2015. Dietary data were collected using a food frequency questionnaire, and protein composition was analysed within a compositional data analysis framework, modelling the balance of AP and PP within the overall macronutrient composition. The associations of protein balances with all-cause mortality were estimated using Cox proportional hazards models adjusted for potential confounders. Effect modification was evaluated through stratified analyses. **Results**: During follow-up, 405 deaths occurred. A greater AP relative to other macronutrients was associated with higher mortality overall (hazard ratio (HR): 1.37; 95% confidence interval (CI): 1.00–1.87) and in males (HR: 1.57; 95% CI: 1.05–2.33). In stratified analyses, these associations were restricted to ever smokers overall (HR 2.06, 95% CI 1.32–3.20), males (HR 1.90, 95% CI 1.18–3.06), females (HR 3.29, 95% CI 1.03–10.54), and to participants with normal weight (HR 1.91, 95% CI 1.07–3.41). No overall association was observed for PP. Among females, PP was associated with lower mortality in those with normal weight. **Conclusions**: The associations between AP and PP and mortality differed by sex, smoking status, and adiposity, supporting more tailored dietary recommendations.

## 1. Introduction

Optimal nutritional balance across the life course is crucial for health and longevity [1], as supported by experimental evidence demonstrating that both caloric intake and dietary macronutrient composition influence lifespan in model organisms [2,3]. In humans, epidemiological studies similarly indicate that macronutrient distributions and overall energy intake predict mortality risk and long-term health outcomes [4,5,6]. Among macronutrients, dietary proteins have received growing attention because of their potential role in shaping health trajectories [7]. In particular, plant-based dietary patterns—typically lower in saturated fats and dietary cholesterol and richer in fibre, antioxidants, and plant proteins—have been associated with a reduced risk of adverse health outcomes, including diabetes, cardiovascular diseases, and cancer [8].

Several prospective cohort studies have examined the association between protein consumption and all-cause mortality, but findings remain inconsistent, particularly when distinguishing between animal protein (AP) and plant protein (PP).

Large cohorts such as the Kuopio Ischaemic Heart Disease Risk Factor Study I [9], the Isfahan Cohort Study [10], and the Nurses’ Health Study & Health Professionals Follow-up Study [11] reported higher mortality associated with AP and lower mortality when PP replaced AP, particularly from red or processed meat. Similar patterns were observed in the US NIH–AARP Diet and Health Study [12]. In contrast, other cohorts—including EPIC Italy [13] and two Japanese prospective studies [14,15], reported null or mixed associations, although substitution analyses often still indicated benefits of PP, particularly among individuals with unhealthy lifestyles or poor dietary patterns. The evidence from pooled analyses further reinforces the importance of protein sources [16,17,18].

Emerging findings also suggest that the health effects of AP and PP vary across subgroups defined by smoking, adiposity, or other lifestyle factors [10,11,13]. However, this evidence remains limited, and potential effect modification has not been systematically investigated.

A major methodological challenge in this field is the compositional nature of dietary data. Because macronutrients are parts of a constrained whole, standard regression approaches for energy adjustment suffer from perfect collinearity and may yield biased or non-interpretable estimates. Substitution models, which omit one or more macronutrients, also have limitations because the interpretation of coefficients depends on the omitted component [19]. To overcome these issues, compositional data analysis (CoDA) has been proposed for modelling nutrient compositions using log-ratio transformations, allowing the estimation of the health effects of specific components while accounting for the relative structure of the diet [20,21,22,23].

To our knowledge, no previous study has applied CoDA to investigate how the relative balance of AP and PP is associated with all-cause mortality. Moreover, evidence on sex-specific associations remains limited [24], and potential modifying roles of smoking and adiposity, strong determinants of mortality, have not been adequately explored. To address these gaps, we examined the association between the relative composition of dietary AP and PP and all-cause mortality in a population-based cohort of middle-aged adults, applying a CoDA framework. We further assessed effect modifications by sex, smoking status, and adiposity.

## 2. Materials and Methods

### 2.1. Study Design, Setting and Population

**Figure 1:**
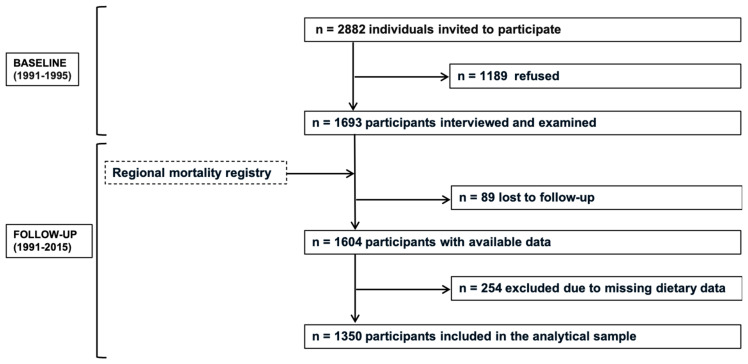
Flow chart describing steps involved in establishing final study sample.

Data were used from a population of individuals aged 40–74 years drawn from the residents’ registry of two districts in Lombardy, Italy. The BEST-FU study methodology has been described previously [25,26]. In brief, the population-based cohort study, conducted from 1991 to 1995, included an in-person examination and the completion of a structured questionnaire. Of the 2882 people initially invited to participate, 1693 agreed, resulting in a response rate of 58.7%. During the hospital visit, participants were asked to complete a series of questionnaires regarding their medical and family history, as well as their past and current drug use and lifestyle habits. They also underwent a general clinical assessment, including anthropometric measurements. In 2015, the health status of participants was retrieved by linking the cohort database with the electronic health records of the regional mortality registries of the Agency for Health Protection (ATS) of Milan (Lombardy Region, Italy), using the fiscal code as a unique identifier. Of the 1604 individuals retrieved from the registries, 1350 (672 males and 678 females) were included in the present analysis after excluding those with unavailable dietary information at baseline (n = 254) (Figure 1).

### 2.2. Dietary Assessment

The study collected information on participants’ typical dietary intake using a quantitative food frequency questionnaire (FFQ)—a modified version of the questionnaire used in the Nurses’ Health Study [27]—which was administered by a trained interviewer. The questionnaire consisted of a list of 158 food and beverage items. The participants were asked to report how often they consumed each item on a seven-level scale ranging from ‘never’ to ‘four to five times per day’ in the year before study entry. To estimate portion sizes, a picture booklet containing photos of foods of three different sizes (small, medium and large) was used. Daily food intake (grams/day) was calculated from the reported frequency of consumption and portion sizes. Reported food intake was then converted into energy and macronutrient intakes, including AP, PP, starch (ST), simple sugars (SSs), saturated fatty acids (SFAs), monounsaturated fatty acids (MUFAs) and polyunsaturated fatty acids (PUFAs), using the Italian food composition database compiled for epidemiological studies [28]. Total energy and energy contribution of each macronutrient were calculated using this database and standard energy conversion factors.

### 2.3. Ascertainment of All-Cause Death

All individuals retrieved from the regional mortality registries of the Agency for Health Protection (ATS) of Milan (Lombardy Region, Italy) were followed up prospectively for all-cause mortality from the start of the study (1 January 1991–1995) until 1 May 2015 (the end of the follow-up period) [25,26].

### 2.4. Covariate Assessment

The physicians conducted interviews with each participant to gather data on their sociodemographic background, personal and family medical history, and past or current medication use and lifestyle-related habits such as physical activity, smoking status, and alcohol consumption. A physical examination was performed at the start of the study to measure body weight and height, from which body mass index (BMI) was calculated (weight (kg)/height (m^2^)) [25]. Educational level was defined as the number of years of formal education completed and categorised into two groups: primary school or less and middle and/or high school or higher. Civil status was categorised as either unmarried (including widowed, separated, divorced, or single) or married. Occupation was classified as retired, employed, homemaker, or other. The participants were categorised as either physically active (engaging in at least one sport per week) or inactive. Smoking status was categorised as never or ever smoking (including former and current smokers). The participants were categorised based on their alcohol consumption as follows: (i) abstainers, (ii) moderate drinkers (≤30 g/day), and (iii) heavy drinkers (>30 g/day).

### 2.5. Dietary Compositional Data Analysis (CoDA)

To take into account the compositional nature of dietary data, i.e., that macronutrients are parts of a whole and therefore convey relative rather than absolute information, macronutrients were analysed using the CoDA approach based on log-ratio transformations [29]. The application of CoDA to dietary data has been extensively discussed in the literature [21,22,23,30,31]. We considered the seven-part macronutrient composition: AP, PP, ST, SS, SFA, MUFA, and PUFA. Our interest was to evaluate the relative dominance of AP and PP in relation to all other macronutrients. Although the *pivot balance* approach is generally used for this purpose, the additive log-ratio (alr) transformation provides equivalent information when estimating the dominance of a given part relative to the others [32]. For a *D*-part composition ($x_{1}$, $x_{2}$, …, $x_{D}$), the alr is defined as:

$$alr\left(x\right)\left[ln\left(\frac{x_{1}}{x_{D}}\right)ln\left(\frac{x_{2}}{x_{D}}\right),...,ln\left(\frac{x_{D-1}}{x_{D}}\right)\right]$$

In our case, six alr coordinates were calculated [30,33] by using ST as the denominator, and each alr term was included as a predictor in the regression model.

Let y be the outcome. The model can be expressed as:

$$f\left(y\right)=B_{0}+B_{1}\cdot ln\left(\frac{AP}{ST}\right)+B_{2}\cdot ln\left(\frac{PP}{ST}\right)+B_{3}\cdot ln\left(\frac{SS}{ST}\right)+B_{4}\cdot ln\left(\frac{SFA}{ST}\right)+B_{5}\cdot ln\left(\frac{MUFA}{ST}\right)+B_{6}\cdot ln\left(\frac{PUFA}{ST}\right)+\sum_{i}B_{i}\cdot z_{i}$$

where $z_{i}$ are the control variables.

The coefficient B_1_, for example, represents the expected change in the outcome when AP increases and ST decreases, while keeping all of the other terms in the model constant. This necessarily means that PP, SS, SFA, MUFA, and PUFA also decrease by the same factor as ST.

To estimate the relative dominance of ST, a second set of six alr coordinates was computed using AP as the denominator, and the regression model was re-estimated accordingly [30]. In the present work, we focused on protein relative dominance, so the results for the other macronutrients are not presented among the main findings but are reported in Supplementary Table S1.

### 2.6. Statistical Analysis

The characteristics of the study population are described using numbers and percentages. Person-years were calculated from baseline until the occurrence of death, loss to follow-up, or the end of the follow-up period, whichever occurred first. A Cox proportional hazards regression model was used to estimate the hazard ratio (HR) and the corresponding 95% confidence intervals (CIs) for the association between alr coordinates and all-cause mortality, using age as the time scale. Schoenfeld’s residuals test confirmed that the ratio of hazards remained proportional and constant over time. HRs and 95% CIs were calculated for all individuals, as well as for males and females. Models were adjusted for birth cohort (in 10-year intervals), educational level, civil status, occupation, smoking status, physical activity, alcohol consumption, daily fibre intake (continuous, in grams) and total daily energy intake excluding alcohol (continuous, in kilocalories). We investigated the potential modifying effects of sex, smoking status (ever vs. never), and adiposity status (using BMI cut-off value of 25 kg/m^2^) on the association between dietary protein and mortality by evaluating stratum-specific estimates. To avoid possible systematic selection of the study population, we performed a sensitivity analysis by comparing the characteristics of the participants included in the analysis (n = 1350) with those who were excluded due to missing dietary information at baseline (n = 254) (Supplementary Table S2). To ensure that the observed associations were not confounded by dietary changes resulting from undiagnosed chronic diseases at baseline, we also repeated the main analysis, excluding deaths that occurred within the first two years of follow-up (n = 16) (Supplementary Table S3). All analyses were conducted using Stata 15 (StataCorp, College Station, TX, USA).

## 3. Results

### 3.1. Description of Cohort

Table 1 shows the baseline characteristics of the 1350 individuals analysed. The mean age of the sample was 57 years (SD 8). Most participants had completed primary school or less, were employed or homemakers, were married, and reported low levels of physical activity. After a median follow-up period of 21 years, 405 deaths were recorded. Compared with females, males were significantly older, better educated, more likely to be employed, married, and more likely to have smoked heavily or consumed alcohol in large quantities. The number of deaths was higher in males than in females: 39% vs. 21%.

**Figure 2:**
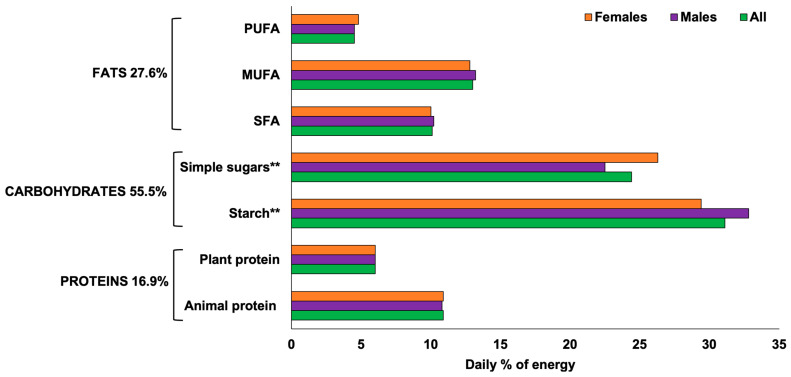
Mean daily % of energy intake from macronutrients. ** *p*-value < 0.001. SFA, saturated fatty acid; MUFA, monounsaturated fatty acid; PUFA, polyunsaturated fatty acid.

Figure 2 shows the daily percentage of energy intake from macronutrients. Proteins represent 16.9% of total energy intake, with 10.9% coming from animal sources and 6% from plant sources. Carbohydrates represent 55.5% of energy intake (31.1% from ST and 24.4% from SS), while total fats represent 27.6% (10% from SFA, 13% from MUFA and 4.5% from PUFA. We observed that males consumed more daily energy, starch and total fat, and fewer SS. No differences in animal or protein % were observed between sexes.

### 3.2. Association Analysis

**Figure 3:**
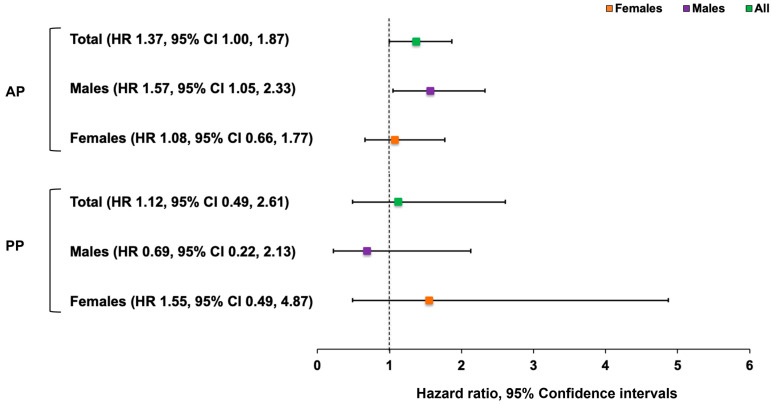
Forest plot for association between AP and PP and mortality. Abbreviations: AP, animal protein; PP, plant protein; BMI, body mass index (kg/m^2^); HR, hazard ratio; CI, confidence interval. Models include terms for birth cohort, educational level, civil status, occupation, smoking status, physical activity, alcohol consumption, daily intake of fibre (g, continuous), and total daily energy intake excluding alcohol intake (Kcal, continuous).

The forest plot (Figure 3) presents the hazard ratios from Cox regression models assessing the association between alr and mortality. In the overall sample, a higher dominance of AP was associated with an increased risk of all-cause mortality (HR = 1.37, 95% CI 1.00, 1.87). Sex-stratified analysis revealed clear effect modifications: AP was associated with increased mortality only in males (HR = 1.57, 95% CI 1.05–2.33), whereas no association was observed in females. There was no overall association with PP, and no sex-specific differences were detected. No other significant associations were observed considering the other macronutrients (Supplementary Table S1).

When we stratified the analysis by smoking status, we observed that AP was associated with a markedly higher mortality risk among ever smokers (current and former). In the total population, ever smokers showed a two-fold higher risk (HR 2.06, 95%CI 1.32, 3.20), with consistent associations in males (HR 1.90, 95% CI 1.18, 3.06) and females (HR 3.29, 95% CI 1.03, 10.54). Stratification by BMI status revealed that AP was significantly associated with increased mortality only among individuals with a BMI < 25 kg/m^2^ (HR 1.91, 95% CI 1.07, 3.41) (Table 2).

Considering PP, sex-stratified analysis revealed a complex pattern, with associations differing markedly by adiposity among females. Specifically, PP was associated with lower mortality among lean females (BMI < 25 kg/m^2^) (HR 0.05, 95%CI 0.003, 0.94), whereas a non-significant trend toward higher mortality was observed among females with BMI ≥ 25 kg/m^2^ (Table 3).

Sensitivity analyses revealed that participants excluded from the analysis were significantly younger, more likely to be male and employed, and more likely to be former smokers and heavier drinkers than those included. However, there was no difference in death status (Supplementary Table S2). To minimise reverse causation due to undiagnosed chronic illnesses, we excluded deaths within the first two years of follow-up, and the results remained essentially unchanged (Supplementary Table S3).

## 4. Discussion

In a cohort of adults living in northern Italy who were followed for more than two decades, shifting the dietary composition toward an increase in animal protein was associated with a 37% increased risk of all-cause mortality. No association was found for plant protein. However, further stratified analysis revealed interaction effects by sex, smoking status, and adiposity, as discussed below.

### 4.1. Animal Proteins

We found that the association with AP was limited to males and to those who were current or former smokers and had a BMI below 25 kg/m^2^, regardless of sex.

Our findings were corroborated by data from previous studies reporting a positive association between AP and mortality, even though they used different approaches to analyse the relationship between protein intake [11,34,35] and mortality. For example, in the Kuopio Ischaemic Heart Disease Risk Factor Study I [9], the authors reported that a higher ratio of animal to plant protein in the diet, as well as higher meat intake, was associated with increased mortality risk. Naghshi et al. [16], in a systematic review and meta-analysis, reported that higher total protein intake was associated with reduced all-cause mortality. Similarly, Chen [6] analysed data from the Rotterdam Study and conducted a meta-analysis of prospective cohorts, concluding that higher AP intake was associated with increased all-cause and cardiovascular mortality. Other recent meta-analyses showed that substitution of AP with PP is associated with lower mortality [17,18].

Consistent with our findings, Haghighatdoost and colleagues [10] demonstrated that increased AP intake was associated with an increased risk of mortality in the Isfahan cohort study, especially among individuals with unhealthy lifestyles such as smoking, heavy drinking, obesity, or physical inactivity. Similarly, in the Nurses’ Health Study & Health Professionals Follow-up Study, Song et al. [11] also reported that high AP intake was linked to increased cardiovascular mortality. In contrast, high PP intake was protective—particularly among individuals with at least one unhealthy lifestyle factor based on smoking, heavy alcohol intake, overweight/obesity, and physical inactivity—with substitution of PP for AP, especially from processed red meat, associated with lower mortality.

Even though food-group analyses were beyond the scope of the present study, it is plausible that the observed association partly reflects the food matrices in which animal proteins are commonly used. In many dietary patterns, animal proteins, particularly when derived from red and processed meat, co-occur with harmful components with pro-oxidant and carcinogenic activities (e.g., *N*-nitroso compounds) [36,37] and that have been linked to increased risks of coronary heart disease, cancer, and mortality. Cigarette smoking represents an additional source of carcinogens and reactive oxidant species. When combined with high animal proteins, smoking may amplify oxidative stress and inflammation, increasing mortality risk [38]. Additionally, smokers often exhibit poorer dietary patterns, including lower intake of antioxidant-rich foods, which reduces their capacity to counteract the oxidative damage generated by certain animal-derived foods. This lack of protection can exacerbate the risk of disease and mortality associated with smoking [39]. A diet rich in animal proteins may further exacerbate this imbalance by increasing the oxidative load without providing compensatory antioxidant protection in smokers [40,41].

### 4.2. Plant Proteins

The divergent associations observed for PP in females, with an increased risk in lean females (BMI < 25 kg/m^2^) and the trend for an increased risk in those with overweight/obesity (BMI ≥ 25 kg/m^2^), suggest that adiposity modifies the metabolic response to plant-derived proteins. To our knowledge, no prior studies have identified these specific associations. However, a cross-sectional study of Indonesian females of reproductive age revealed a positive correlation between higher PP intake (mainly from grains) and BMI [42]. In contrast, a study of Australian adults found that PP intake was inversely associated with BMI and waist circumference in males but not in females after adjusting for energy intake [43]. Compared with lean females, individuals with overweight/obesity are characterised by chronic low-grade inflammation, insulin resistance, and an altered metabolic profile. We speculate that these conditions influence the way dietary proteins and other nutrients are processed, potentially modifying the physiological response to plant-based diets. As a result, the benefits generally attributed to higher plant protein intake may be attenuated in females with excess adiposity [44]. Another possible explanation is interindividual variability in gut microbiota function, which affects the biotransformation of the non-protein compounds present in plant-based foods (e.g., phytoestrogens). Differences in microbial metabolism have been linked to variability in cardiometabolic risk, and some evidence suggests that such variability is more pronounced among individuals with overweight or obesity, limiting the potential benefits of plant-rich diets in this group [45,46]. In addition, plant-based diets might mitigate the genetic risk of obesity on cardiometabolic markers more effectively in genetically susceptible females [47]. However, because this subgroup analysis involved relatively small numbers of participants and deaths, resulting in imprecise estimates with wide confidence intervals, these findings should be interpreted cautiously and considered hypothesis-generating.

### 4.3. Strengths and Limitations

Notably, there are several limitations in interpreting our findings. Dietary intake was assessed only once at baseline and was assumed to reflect habitual midlife intake. Consequently, changes in dietary habits over time may have led to non-differential exposure misclassification for both absolute intake and macronutrient composition. Such misclassification may attenuate the associations between macronutrient and mortality, particularly if the relative contributions of animal proteins, plant proteins, and other macronutrients changed over time, thereby biasing the estimates toward the null.

Furthermore, selection bias is a potential concern, as excluding slightly younger, male, employed, smokers and alcohol drinkers participants may limit the generalisability of our findings. However, the exclusion of higher-risk individuals may have attenuated the associations and shifted the results towards the null.

Furthermore, residual measured or unmeasured confounding cannot be entirely ruled out because of factors not considered in this analysis (e.g., comorbidities, medications, broader socioeconomic indicators, and changes in lifestyle over time), despite controlling for several potential risk conditions.

Finally, the borderline association of PP with increased mortality observed in females with overweight/obesity may be partly explained by reverse causation, as those with conditions such as diabetes or cardiovascular disease often adopt such dietary patterns on medical advice. In such cases, mortality risk would likely reflect the underlying illness rather than diet itself. By contrast, in lean females, a higher PP balance may simply reflect a generally healthier lifestyle, including greater consumption of vegetables and higher physical activity. However, because the associations remained after excluding early deaths and were independent of fibre intake and other lifestyle factors, reverse causation is unlikely to fully explain our findings.

One of the strengths of this study is its use of the CoDA approach to study the relationship between protein balance and mortality, a technique that has not previously been used to examine this association. Unlike traditional regression, which struggles with the compositional nature and collinearity of macronutrients, often yielding inconsistent or spurious results depending on which nutrient is excluded, CoDA treats nutrients in relation to one another. This enables proportional changes to be estimated without distorting their compositional balance.

Further strengths of this study include its prospective, population-based design, which limits the potential for reverse causation, and its long follow-up period. Additionally, detailed information on potential risk factors for mortality was collected at baseline, enabling rigorous adjustment for confounding factors in the analysis.

## 5. Conclusions

This study provides the first compositional analysis of dietary protein in relation to mortality, suggesting that the balance between protein sources plays a role in long-term health. By applying a compositional data analysis framework, our findings highlight the importance of evaluating dietary components in relation to one another rather than in isolation, offering a more comprehensive understanding of dietary effects.

Although the sex-, adiposity-, and smoking-specific associations we observed are novel, they should be interpreted with caution. Future longitudinal and experimental studies in different populations are needed to confirm our results.

From a public health perspective, however, these findings indicate that the balance of dietary protein sources could be relevant for implementing more targeted preventive strategies aimed at reducing long-term adverse health outcomes, particularly among individuals with less-healthy behaviours.

## Figures and Tables

**Table 1:** Baseline participants’ characteristics by sex.

	All (n = 1350)	Males (n = 672, 49.8%)	Females (n = 678, 50.2%)	*p*-Value
Baseline Characteristics	n (%)	n (%)	n (%)	
Birth cohort (calendar years)				0.006
<1920	107 (7.9)	70 (10.4)	37 (5.5)	
1920–1929	388 (28.7)	191 (28.4)	197 (29.1)	
1930–1939	611 (45.3)	300 (44.6)	311 (45.9)	
≥1940	244 (18.1)	111 (16.5)	133 (19.6)	
Age (mean, SD)	57 (8)	58 (8.3)	57 (7)	0.050
Educational level				<0.001
Primary school or less	773 (57.3)	331 (49.3)	442 (65.2)	
Middle/high school or graduate	577 (42.7)	341 (50.7)	236 (34.8)	
Occupation				<0.001
Retired	531 (39.3)	307 (45.7)	224 (33.0)	
Employed	467 (34.6)	300 (44.6)	167 (24.6)	
Homemakers	240 (17.8)	1 (0.2)	239 (35.3)	
Other	112 (8.3)	64 (9.5)	48 (7.1)	
Civil status				<0.001
Unmarried	202 (15.0)	52 (7.7)	150 (22.1)	
Married	1148 (85.0)	620 (92.3)	528 (77.9)	
BMI (mean, SD)	27.02 (4.1)	26.9 (3.4)	27.12 (4.7)	0.362
BMI < 25 kg/m^2^	447 (33.1)	198 (29.5)	249 (36.7)	0.005
BMI ≥ 25 kg/m^2^	903 (66.9)	474 (70.5)	429 (63.3)	
Weekly physical activity				0.073
Inactive	1085 (80.4)	527 (78.4)	558 (82.3)	
Active	265 (19.6)	145 (21.6)	120 (17.7)	
Smoking status				<0.001
Never smokers	695 (51.5)	197 (29.3)	498 (73.5)	
Ever smokers	655 (48,5)	475 (70.7)	180 (26,8)	
Energy intake (Kcal)	3022.9 (2839.8)	3329.7 (1295.3)	2718.8 (989.8)	<0.001
Alcohol consumption				<0.001
Abstainer	407 (30.2)	89 (13.2)	318 (46.9)	
Moderate drinkers	298 (22.1)	178 (26.5)	120 (17.7)	
Heavy drinkers	645 (47.8)	405 (60.3)	240 (35.4)	
Deaths	405 (30)	262 (39.0)	143 (21.1)	<0.001

Abbreviation: BMI, body mass index (kg/m^2^). Numbers are frequencies and percentages if unless specified.

**Table 2:** Hazard ratios for all-cause mortality associated with animal protein (alr), stratified by smoking and adiposity.

	Males (n = 672, 49.8%)	Females (n = 678, 50.2%)	All (n = 1350)
Animal Protein	HR (95% CI)	HR (95% CI)	HR (95% CI)
Smoking habit			
Never smokers	0.91 (0.40, 2.06)	0.80 (0.47, 1.37)	0.86 (0.55, 1.35)
Ever smokers	1.90 (1.18, 3.06)	3.29 (1.03, 10.54)	2.06 (1.32, 3.20)
BMI status			
BMI < 25 kg/m^2^	1.38 (0.68, 2.79)	2.57 (0.92, 7.20)	1.91 (1.07, 3.41)
BMI ≥ 25 kg/m^2^	1.62 (0.97, 2.71)	0.75 (0.42, 1.33)	1.22 (0.83, 1.78)

Abbreviation: BMI, body mass index (kg/m^2^); HR, hazard ratio; CI, confidence interval. Models include terms for birth cohort, educational level, civil status, occupation, smoking status, physical activity, alcohol consumption, daily intake of fibre (g, continuous), and total daily energy intake excluding alcohol intake (Kcal, continuous).

**Table 3:** Hazard ratios for all-cause mortality associated with plant protein (alr), stratified by smoking and adiposity.

	Males (n = 672, 49.8%)	Females (n = 678, 50.2%)	Total (n = 1350)
Plant Protein	HR (95% CI)	HR (95% CI)	HR (95% CI)
Smoking status			
Never smokers	0.76 (0.11, 5.02)	1.49 (0.42, 5.26)	1.43 (0.49, 4.21)
Ever smokers	1.14 (0.29, 5.28)	0.98 (0.08, 12.07)	1.05 (0.31, 3.53)
BMI status			
BMI < 25 kg/m^2^	1.06 (0.14, 8.22)	0.05 (0.003, 0.94)	0.47 (0.09, 2.56)
BMI ≥ 25 kg/m^2^	0.63 (0.18, 2.20)	3.06 (0.91, 10.28)	1.37 (0.56, 3.33)

Abbreviation: BMI, Body mass Index (kg/m^2^); HR, hazard ratio; CI, confidence intervals. Models include terms for birth cohort, educational level, civil status, occupation, smoking status, physical activity, alcohol consumption, daily intake of fibre (g, continuous), and total daily energy intake excluding alcohol intake (Kcal, continuous).

## Data Availability

The data described in the manuscript, code book, and analytic code will be made available upon request of pending application and approval.

## Supplementary Materials

The following supporting information can be downloaded at: https://www.mdpi.com/article/10.3390/nu18122035/s1, Table S1: Hazard ratios for all-cause mortality associated with remaining macronutrients. Table S2: Characteristics of the study population included and excluded from the analysis (n = 1604); Table S3: Hazard ratios for all-cause mortality associated with animal and plant protein (alr), stratified by smoking and adiposity after exclusion of participants who died within 2 years of follow-up (n = 16).

## Abbreviations

The following abbreviations are used in this manuscript:

CoDA	Compositional data analysis
BEST-FU	Bollate Eye Study Follow-up
ATS	Agency for Health Protection
FFQ	Food-frequency questionnaire
AP	Animal protein
PP	plant protein
ST	Starch
SS	Simple sugar
SFA	Saturated fatty acids
MUFA	Monounsaturated fatty acids
PUFA	Polyunsaturated fatty acids
BMI	Body mass index
alr	Additive log ratio
HR	Hazard ratio
CI	Confidence interval
