# Supplementary material for: Association of Dietary Animal and Plant Protein Composition with All-Cause Mortality: 24-Year Population-Based Cohort Study
Source: Nutrients. 2026 Jun 22;18(12):2035. doi: 10.3390/nu18122035 (PMC13304937; doi:10.3390/nu18122035)
Supplement: Supplementary file 1 [file nutrients-18-02035-s001.zip › nutrients-4355445-supplementary.pdf]

**Supplementary Table S1. Hazard ratios for all-cause mortality associated with the remaining macronutrients.**

| | **Males** | **Females** | **Total** |
|---|---|---|---|
| **Other macronutrients** | **HR (95% CI)** | **HR (95% CI)** | **HR (95% CI)** |
| Simple sugars vs starch | 0.92 (0.65-1.28) | 1.09 (0.69-1.71) | 0.98 (0.74-1.29) |
| Starch vs animal protein° | 1.36 (0.56-3.31) | 0.71 (0.33-1.51) | 0.90 (0.48-1.67) |
| SFA vs starch | 0.71 (0.39-1.29) | 0.92 (0.42-2.03) | 0.75 (0.46-1.23) |
| MUFA vs starch | 1.10 (0.62-1.95) | 0.52 (0.23-1.20) | 0.86 (0.54-1.39) |
| PUFA vs starch | 0.95 (0.64-1.42) | 1.62 (0.95-2.75) | 1.14 (0.83-1.57) |

Abbreviations: HR, hazard ratio; CI, confidence intervals; BMI, body mass index; SFA, saturated fatty acids; MUFA, monounsaturated fatty acids; PUFA, polyunsaturated fatty acids.

Models include terms for years of age, educational level, civil status, occupation, smoking status, physical activity, alcohol consumption, daily intake of fibre (g, continuous), and total daily energy intake (Kcal, continuous). °Obrained in a second model that included the six alr with animal protein as the denominator.

**Supplementary Table S2. Characteristics of the study population included from excluded form the analysis (n=1604)**

| | **Included (n=1350)** | **Excluded (n=254)** | |
|---|---|---|---|
| | **n (%)** | **n (%)** | ***p-value*** |
| Sex | | | <0.001 |
| Males | 672 (49.78) | 194 (76.38) | |
| Females | 678 (50.22) | 60 (23.62) | |
| Age at baseline, years (mean, SD) | 57.35 (8.09) | 55.89 (6.95) | 0.002 |
| Educational level | | | 0.831 |
| Primary school or less | 773 (57.26) | 147 (57.87) | |
| Middle school | 341 (25.26) | 60 (23.62) | |
| High school or graduate | 236 (17.48) | 47 (18.50) | |
| Occupation | | | <0.001 |
| Retired | 531 (39.33) | 95 (37.40) | |
| Employed | 467 (34.59) | 131 (57.57) | |
| Housewife | 240 (17.78) | 23 (9.06) | |
| Other | 11 (8.30) | 5 (1.97) | |
| Married | 1148 (85.04) | 216 (85.04) | 0.999 |
| BMI (mean, SD) | 27.02 (4.10) | 26.80 (3.75) | 0.067 |
| Weekly physical activity | 265 (19.63) | 60 (23.62) | 0.146 |
| Smoking habit | | | |
| Never smokers | 695 (51.48) | 98 (38.58) | |
| Ever smokers | 655 (48.52) | 156 (61.42) | <0.001 |
| Alcohol consumption | | | <0.001 |
| Abstainer | 407 (30.15) | 52 (20.47) | |

| | | | |
|---|---|---|---|
| Moderate drinkers | 298 (22.07) | 28 (11.02) | |
| High drinkers | 645 (47.78) | 174 (68.50) | |
| All-cause death | 405 (30) | 70 (27.56) | 0.434 |

Abbreviation: SD, standard deviation; BMI, Body mass index (Kg/m$^2$), Numbers are frequencies and percentages if unless specified.

**Table S3. Hazard ratios for all-cause mortality associated with animal and plant protein (alr), stratified by smoking and adiposity after exclusion of participants who died within 2 years of follow-up (n=16)**

| | **Males** | **Females** | **Total** |
|---|---|---|---|
| | **HR (95% CI)** | **HR (95% CI)** | **HR (95% CI)** |
| **Animal protein** | 1.54 (1.03, 2.31) | 1.11 (0.67, 1.85) | 1.38 (1.00, 1.89) |
| Smoking habit | | | |
| Never smokers | 0.91 (0.40-2.05) | 0.82 (0.47-1.43) | 0.88 (0.56, 1.39) |
| Ever smokers | 1.87 (1.15-3.05) | 3.40 (1.06, 10.93) | 2.07 (1.32, 3.24) |
| BMI status | | | |
| BMI < 25 Kg/m$^2$ | 1.27 (0.62-2.58) | 2.60 (0.91, 7.45) | 1.78 (0.99, 3.21) |
| BMI ≥ 25 Kg/m$^2$ | 1.63 (0.97-2.73) | 0.77 (0.43, 1.39) | 1.24 (0.84, 1.83) |
| **Plant protein** | 0.60 (0.19, 1.90) | 1.49 (0.46, 4.79) | 1.10 (0.47, 2.59) |
| Smoking habit | | | |
| Never smokers | 0.75 (0.11-5.26) | 1.37 (0.37, 5.00) | 1.39 (0.46, 4.15) |
| Ever smokers | 0.91 (0.22-3.70) | 1.01 (0.08, 12.24) | 0.93 (0.27, 3.20) |
| BMI status | | | |

| | | | |
|---|---|---|---|
| BMI < 25 Kg/m$^2$ | 1.00 (0.12-8.17) | 0.05 (0.003, 0.86) | 0.51 (0.09, 2.87) |
| BMI ≥ 25 Kg/m$^2$ | 0.54 (0.15-4.91) | 2.81 (0.81, 9.71) | 1.29 (0.52, 3.20) |

Abbreviations: HR, hazard ratio; CI, confidence intervals; BMI, body mass index.

Models include terms for years of age, educational level, civil status, occupation, smoking status, physical activity, alcohol consumption, daily intake of fibre (g, continuous), and total daily energy intake (Kcal, continuous).
